# Endothelial Glycocalyx Injury in SARS-CoV-2 Infection: Molecular Mechanisms and Potential Targeted Therapy

**DOI:** 10.1155/2023/6685251

**Published:** 2023-08-29

**Authors:** Bingxuan Lv, Shengshi Huang, Hong Huang, Na Niu, Ju Liu

**Affiliations:** ^1^The Second Hospital of Shandong University, Shandong University, 247 Beiyuan Street, Jinan 250033, China; ^2^Medical Research Center, Shandong Provincial Qianfoshan Hospital, Shandong University, 16766 Jingshi Road, Jinan 250014, China; ^3^Department of Pediatrics, Shandong Provincial Hospital, Shandong First Medical University, 324 Jingwu Road, Jinan 250021, China

## Abstract

This review aims at summarizing state-of-the-art knowledge on glycocalyx and SARS-CoV-2. The endothelial glycocalyx is a dynamic grid overlying the surface of the endothelial cell (EC) lumen and consists of membrane-bound proteoglycans and glycoproteins. The role of glycocalyx has been determined in the regulation of EC permeability, adhesion, and coagulation. SARS-CoV-2 is an enveloped, single-stranded RNA virus belonging to *β*-coronavirus that causes the outbreak and the pandemic of COVID-19. Through the respiratory tract, SARS-CoV-2 enters blood circulation and interacts with ECs possessing angiotensin-converting enzyme 2 (ACE2). Intact glycolyx prevents SARS-CoV-2 invasion of ECs. When the glycocalyx is incomplete, virus spike protein of SARS-CoV-2 binds with ACE2 and enters ECs for replication. In addition, cytokine storm targets glycocalyx, leading to subsequent coagulation disorder. Therefore, it is intriguing to develop a novel treatment for SARS-CoV-2 infection through the maintenance of the integrity of glycocalyx. This review aims to summarize state-of-the-art knowledge of glycocalyx and its potential function in SARS-CoV-2 infection.

## 1. Introduction

The endothelial glycocalyx is a dynamic hair-like network layer composed of protein and polysaccharide that covers the luminal surface of endothelial cells (ECs) and acts as a barrier between blood and vascular walls. Under normal conditions, the thickness of microvessel glycocalyx varies with different tissues and species, ranging from less than 100 nm to about 1 *μ*m [1]. For example, in vivo studies have revealed that in muscle capillaries, the glycocalyx thickness is about 0.5 *μ*m thick [2]. It has also been shown that in the arterial system, glycocalyx's thickness increases with vascular diameter, ranging from 2 to 3 *μ*m in small arteries [3].

## 2. Structure, Composition, and Function of Glycocalyx

The main functional components in the glycocalyx are proteoglycans (PGs) and associated glycosaminoglycan (GAG) side chains. According to the properties of glucose residues, sulfation level, and so on, GAG is classified into chondroitin sulfate (CS), dermatan sulfate (DS), heparan sulfate (HS), hyaluronic acid (HA), keratan sulfate, and heparin. The carboxyl groups of sulfate and aldehyde acid are mostly negatively charged and tend to bind to positive metal ions, thus showing hydrophilicity. HS is regarded as the most prominent component in over 50% proportion. Under normal physiological conditions, the HS to CS ratio remains consistently around 4 : 1. However, this ratio undergoes alterations during pathological conditions [4, 5]. The cell surface PG found in mouse mammary epithelial cells contains GAGs, namely CS and HS.

PGs are intricate macromolecules composed of a core protein that is covalently adorned with sulfated GAG chains, which vary in size and structure. However, each core protein bears more than one type of GAG chain. PGs exhibit differences in the dimensions of the core proteins and the abundance of GAG side chains, as well as their interactions with the cell membrane. With the aid of link protein, abundant PG monomers and HA make up syndecan (SDC), whose core proteome is firmly connected to the cell membrane via transmembrane domains [6]. The SDC family belongs to the transmembrane PGs found in the glycocalyx, where the core protein groups of glypicans are connected firmly through glycosylphosphatidylinositol anchor to the cell membrane [7]. Various core proteins, including biglycans, versicans, and mimecans, carry DS or CS as part of their PGs [8]. Perlecan, present in the basement membrane, is a substantial PG with HS [7]. SDC is released in a soluble form when the glycocalyx becomes disrupted. In addition to this, the other PGs, such as soluble perlecan, are found within the glycocalyx or disperse into the bloodstream upon secretion [9]. The glycocalyx demonstrates physiological activity when plasma constituents bind to or interact with it [5].

Glycocalyx cannot be described as a static structure because there is a dynamic equilibrium between the biosynthesis and shedding of glycocalyxes; in particular, HA is turned over rapidly [10, 11]. For the diffusion of soluble components into the bloodstream, it indicates that there is no clear boundary between the glycocalyx and the bloodstream. The composition and sizes wave with changing shear rates from turbulent blood, resulting in the absence or rebuild of glycocalyx constituents [10]. The production of the glycocalyx involves a variety of signaling pathways [12]. Several factors, including local pH and mechanical stress, regulate its inactivation [5, 13].

The endothelial glycocalyx participates in many processes because of its structure and location, such as mechanotransduction, the regulation of vascular permeability, the theological behavior of the microcirculation, the binding of blood cells to the endothelium, and anticoagulation. The perturbation of the endothelial glycocalyx participates in varieties of pathophysiological sequelaes, such as pneumonia.

The endothelial glycocalyx regulates vascular permeability to control the shift of albumin and other circulating plasma components (mainly other proteins) across the endothelium [14]. Reactive oxygen species (ROS), matrix metalloproteinases, and heparanase are activated to cause variable glycocalyx density and thickness during inflammation.

In vitro models, ECs produce glycocalyx to regulate permeability. In vivo models, for example, in guinea pig heart, researchers found that using ischemia or histamine to cause cellular barrier disruption is as effective as using heparinase to induce glycocalyx damage in terms of increasing coronary vessel leakage, which leads to the hypothesis that different kinds of cellular glycocalyx damage results in different degrees of vessel permeability [15]. Under pathological conditions of ischemia and hypoxia, significant abscission of lung polysaccharide calyx in ECs is induced, leading to a significant increase in vascular permeability [16]. Moreover, when researchers focus on the glomerular filtration barrier (GFB), recent models of GFB suggest that glycocalyx represents a significant protein barrier. The block of protein leakage is the biological function reflecting vessel permeability [17]. According to researchers' measurements, different molecules on endothelial surface layer lead to different permeability of the EC surface layer. The result implies that multiple factors may affect the penetration of the barrier, including molecular size, charge, and configuration [18].

Glycocalyx promotes white blood cell (WBC)–EC adhesion by HS and HA shedding during an inflammatory process. In vivo, it has been found that shedding of the endothelial glycocalyx is in response to inflammation [19]. Furthermore, in an animal model of inflammation, the glycocalyx experiences rapid shedding of glycans and reduced thickness. These changes facilitate the penetration of WBCs and their adhesion to the ECs [1]. Other findings show that inflammation initiates endothelial apoptosis. As a consequence, glycocalyx comes to shedding and finally facilitates monocyte adhesion and macrophage infiltration. The outermost layer, primarily composed of HS PGs, may be easily penetrated by WBC. The glycocalyx provides minimal resistance to microvilli on the rolling WBCs' surface until they reach a deeper layer of hyaluronan. Upon activation by inflammatory mediators, the endothelium may shed its first component, which could be the compactly located HA near the surface of the EC. Consequently, enzymatic shedding of HA could significantly boost the firm adhesion between WBCs and ECs. As the layer becomes more porous, it facilitates rapid WBC–EC firm adhesion by making adhesion receptors more accessible [20, 21].

The glycocalyx translates biomechanical forces into biochemical signals. The EC glycocalyx contains an HS component that plays a role in mechanosensing, facilitating nitric oxide (NO) production in response to shear force [22]. After that, it is proven that HS plays a major role in mechanosensing [23]. The HS PG glypican-1 serves as the main mechanosensor responsible for the production of NO under shear-induced conditions [24]. Exposing to shear stress, ECs produce NO [25]. NO synthases are a family of complex cytochrome P45o-like hemeproteins that catalyze to form NO [26]. Upon receiving a biochemical signal, the concentration of calcium ions increases sharply, producing a large amount of calmodulin, activating NO synthase to produce NO [27]. NO diffuses rapidly and isotropically through ECs. NO's swift diffusion between cells enables it to effectively coordinate blood vessel responses to turbulence within a localized region [28]. To testify glycocalyx mechanotransducer function on a molecular level, for example, treatment to selectively break down HS GAGs leads to insufficient responses to shear variations and damaged NO production [22].

Glycocalyx sustains the blood flow through preventing the formation of a thrombus and keeping the amount of anticoagulant on the glycocalyx. On the one hand, as a physical barrier, it prevents platelets from contacting with ECs. Circulating platelets in the bloodstream contact with EC glycocalyx directly. The intact endothelium possesses a variety of anticoagulant properties, which involve the generation and liberation of prostacyclin, NO, and tissue factor pathway inhibitor (TFPI) [29]. In pathological conditions, after initiating the coagulation pathway, platelets adhere to damaged endothelium tightly via platelet surface glycoproteins GPIb alpha, GPIIb/IIIa [30], and GPIIb/IIIa [31]. P-selectin, vWF, ICAM-1, and PECAM-1 promote coagulation. P-selectin expresses on activated platelet surface and mediates platelet rolling on EC [32]. According to a previous experiment, P-selectin binds with PSGL-1 to regulate the amount of tissue factor in the blood to regulate the coagulation process. Furthermore, as a cell adhesion molecule on microparticles, PSGL-1 delivers tissue factor to the developing thrombus [33]. vWF connects platelet GPIb-IX complex to subendothelial collagen to mediate platelet adhesion. Studies have documented that ICAM-1 promotes a GPIIb/IIIa-dependent bridging mechanism involving fibrinogen [30]. PECAM-1 inhibits platelet signaling and function derived by various receptors, including the GPVI, GPIb-V-IX complex, and Fc gamma RIIA [34]. In addition, it also promotes platelet EC adhesion at injury sites [35]. On the other hand, GAG of glycocalyx binds with anticoagulants, including thrombin III, TFPI, and thrombomodulin (TM), which means the loss of GAG leads to the loss of anticoagulant. Antithrombin III binds with HS to enhance its anticoagulant activity [36]. It inhibits thrombin and activated factors X and IX [37]. Together with tissue factor and blood protease factors, TFPI creates a compound to inhibit thrombin generation and fibrin formation. TM, an endothelium-bound protein, possesses anticoagulant and anti-inflammatory properties and gets activated in response to the stimulation of procoagulants [38]. TM binds to CS and converts thrombin to activate the protein C pathway to anticoagulation [39], while TFPI inhibits FVIIa and FXa. Mostly, TFPI binds to the glycocalyx via HSs, but some proteins attend that [40]. After the destruction of the glycocalyx, anticoagulant substances, such as antithrombin III (AT III), heparin cofactor II, and TM, are depleted. This leads to an imbalance between procoagulant and anticoagulant factors [41].

## 3. Structure of SARS-CoV-2 and Clinical Symptoms of COVID-19

The initial emergence of the COVID-19 outbreak was documented in December 2019, in the city of Wuhan, China. The pathogen, SARS-CoV-2, belongs to the Nidovirales order, the Coronaviridae family, and the *β*-coronavirus genus [42]. Previous studies indicated that betacoronavirus is an enveloped, single-stranded RNA virus capable of infecting wild animals, herds, and human beings, leading to infections without apparent symptoms or even occasional outbreaks [43]. Until now, the SARS-CoV-2 variant of concern includes the alpha, beta, delta, and omicron [44, 45].

The virus genome is single-stranded positive RNA, which codes nucleoprotein (N), membrane protein (M), envelop protein (E), spike glycoprotein (S), and polymerase (Pol). Coronaviruses consist of essential structural elements, including the glycoprotein S, the transmembrane proteins M and E, and the nucleoprotein N, which combines with the viral RNA to create a viral ribonucleoprotein (RNP) complex [46]. Virus is sensitive to ether, trichloromethane, esters, and ultraviolet. It loses its infectivity within a few hours at 37°C.

The genome structure of SARS-CoV-2 follows the characteristic organization found in *β*-coronaviruses with sequences similar to many coronaviruses, including bat coronavirus RaTG13, SARS-CoV, and so on. Among them, SARS-CoV has 79% similarity with SARS-CoV-2. It indicates that SARS-CoV is the closest relative of SARS-CoV-2 among human coronaviruses [42]. Bat coronavirus RaTG13 has 98% similarity with SARS-CoV-2. Reviewing researchers' comparison between the sequence of SARS-CoV-2 and bat coronavirus, it is found that it shared a 96.2% identity with RaTG13 [47]. Also, coronavirus sequences in the pangolin also share high similarity with SARS-CoV-2 [48].

Previous research suggests that spike protein binding to the angiotensin-converting enzyme 2 (ACE2), whose host target receptor is expressed by various cell types in the lung, is the primary step of infection [49]. In addition to ACE2, SDCs are utilized as coreceptors of internalization. Members of the SDC family exhibit tissue-specific expression patterns: SDC1 is found on epithelial and plasma cells, SDC2 is present on ECs, SDC3 is located on neurons, and SDC4 is widely distributed throughout various tissues [50–53]. According to the BioGPS gene expression database, SDC4 shows significant expression in human lung cells [53, 54].

As the coronavirus spreads across the globe, evidence is mounting that many people infected with COVID-19 are asymptomatic, yet they can spread the virus to others. Asymptomatic infections denote cases where SARS-CoV-2 nucleic acid is detected in patient samples through reverse transcriptase-polymerase chain reaction. These individuals do not exhibit typical clinical symptoms; however, their imaging resulting, including lung computed tomography (CT), show no apparent lesions [55]. According to data extracted from patients with laboratory-confirmed COVID-19, researchers found that the main symptoms are fever (≥38°C), cough, myalgia or fatigue, and lymphocytopenia. More than half of patients suffer from dyspnea. Nausea, vomiting, sputum production, headache, hemoptysis, and diarrhea are uncommon, and most CT scan results show abnormal results. Ground-glass opacity and bilateral patchy shadowing are the prevailing patterns observed on chest CT scans [56]. Severe COVID-19 cases progress to acute respiratory distress syndrome (ARDS), on average, about 8–9 days after the onset of symptoms. Overall, 70% ARDS leads directly to respiratory failure, then progress to lethal COVID-19 cases [57]. In addition, irreformable blood coagulation disorders occur [58]. Most patients have mild symptoms and a good prognosis. After infection, a protective antibody is produced to protect the human body temporarily. On account of the immunological memory is not strong, reinfection occurs sometimes.

Sources of infection include patients and asymptomatic carriers, in addition to the virus considering wildlife as hosts, such as pangolin and *Rhinolophus sinicus*. Animal-to-human transmission is one of the means of causing human infection [59]. COVID-19 primarily spreads through respiratory particles, and it is well-established that presymptomatic, paucisymptomatic, and asymptomatic individuals can transmit the virus [60]. Like the other respiratory coronaviruses, SARS-CoV-2 is transmitted primarily through respiratory droplets, although the cases of close contact transmission route occur occasionally. Judging from the cases of patients, there is clear evidence to prove that human-to-human transmission is indeed effective among close contacts. While SARS-CoV-2 RNA diminishing in respiratory and stool samples can persist for an extended period, the duration of viable virus is relatively brief. The SARS-CoV-2 titers in the upper respiratory tract reach their highest levels during the first week of illness [61]. Depending on the characteristics of the patient, older people are more susceptible than others, while male patients are generally more affected than women [62]. The median incubation period, starting from the onset of symptoms, lasted approximately 4–5 days [63].

## 4. The Process of Viral Direct Invasion and Cytokine Storm

Based on the clinical data, it shows that in critically ill patients with COVID-19, the occurrence of endothelial damage involves disruption of glycocalyx integrity [64]. Due to the negative charge from the HS component of the glycocalyx, glycocalyx interacts electrostatically with the viral *S*-proteins by binding with the positively charged domain of *S*-protein [65]. In the meantime, since the thickness of the glycocalyx, direct contact with ACE2 is prohibited. PG core protein interacts with HS to avoid virus attachment [66]. HS binds with core protein at Ser residues. Competitively, SARS-CoV-2 is glycosylated at HS attachment sites. To some extent, the sequences inhibit virus internalization and glycocalyx damage [67]. Furthermore, the unharmed endothelial glycocalyx possesses specific attachment locations for antioxidant enzymes such as xanthine oxidoreductase and endothelial superoxide dismutase [68, 69]. So, it means healthy glycocalyx quencher free radicals. Alternatively, when SARS-CoV-2 attacks intact glycocalyx, glycocalyx gets thicker to protect itself. Besides, especially for someone who have an allergic physique, an allergic physique is associated with lower infection risk [70–72]. The likelihood of severe COVID-19 in individuals with asthma and other allergic conditions may be lower due to diminished ACE2 gene expression in airway cells, resulting in reduced vulnerability to infection [73]. Moreover, persistent inflammation in asthmatic lungs might induce a level of immune tolerance that, in effect, limits the progression of the exaggerated inflammatory reaction responsible for the severity of COVID-19 [74]. Excessive mucus production in asthma patients serves as a barrier, preventing SARS-CoV-2 from reaching the alveolar type 2 cells, which are the primary cells expressing ACE2 in the lungs [75, 76].

However, under abnormal conditions, after SARS-CoV-2 enters the lower respiratory tract from the environment, viral spike protein prefers sialylated glycans with alpha-(2,6)-sialic acids on the termini positions. Consequently, it is probable that SARS-CoV-2 interacts with cells and tissues abundant in sialylated glycans, including N─, O─, and possibly glycolipids situated on the epithelial surface [77]. According to previous research, ACE2 and HS of EC glycocalyx contain sialic acid, which can facilitate the interaction between virus and host cells. Virus establishes connections with host cells by exploiting its target receptors ACE2 and HS. In the process of specific binding between ligand and receptor, glycocalyx changes. HS functions as an initial host attachment factor that facilitates SARS-CoV-2 infection. HS directly binds to Spike, thereby aiding the attachment of Spike-bearing viral particles to the cell surface by interacting with the glycocalyx [78]. In terms of mechanisms, viruses take advantage of the HS interaction to enhance their concentration on the cell surface and increase their likelihood of encountering a more specific entry receptor [79–81]. The presence of a virus particle on the cell surface amplifies the millimolar affinities for a monovalent protein–glycan interaction through multivalency/avidity arising from numerous low-affinity binding sites. This leads to the establishment of biologically significant interactions [82]. The SARS-CoV-2 efficiently uses GAGs on the glycocalyx for initial attachment by low-affinity, high-avidity interactions, and then the high-affinity interaction of the spikes with the ACE2 receptor occurs [83]. Before the fusion between virus and target cells, spike receptor binding domain (RBD) adopts two kinds of conformations, including up and down positions [65, 84]. HS binds with spike in spite of RBD conformation [65]. However, unlike HS, ACE2 requires upconformation only to interact with spikes [85–87]. To cause the subunit S1-RBD to undergo a conformational change into the open conformation, the spike protein interacts with cell surface HS, resulting in an increased number of RBDs in the “up/open” conformation, consequently enhancing binding to ACE2 receptors [65]. Specific interactions between spike protein and ACE2 increase, then stabilize and enhance the invasion [88].

Moreover, in parallel with the pathway above, given the SDC-centered pathway's existence, viruses bind to the core proteins first. From the exterior, glycocalyx is incomplete, while from the inside, using core proteins as receptors and S1 subunit as ligands, viruses enter the cell by endocytosis. And the endocytosis is accompanied with the endocytic degradation of core proteins [89]. Virus spike S1 connects with SDC ectodomain first. The binding of SARS-CoV-2 to the SDC4 ectodomain is not solely governed by the HS chains; it is also influenced by other components of the SDC ectodomain, including SDC4's cell-binding domain [90]. In SDC-mediated endocytosis, the clustering of SDCs induced by ligands leads to the repositioning of SDCs to lipid rafts. This, in turn, triggers a lipid raft-dependent internalization of the SDC-ligand complex, which occurs independently of clathrin and caveolae [89, 91, 92].

The role of ACE2 is to promote the hydrolysis of angiotensin II (Ang II) to produce angiotensin 1 to angiotensin 7. It acts on Mas receptors, dilates vessels, and protects lung tissues against inflammation [93]. For incomplete glycocalyx, the structural barrier formed by the glycocalyx is impaired, and ACE2 expression is upregulated. When SARS-CoV-2 Spike protein binds to ACE2, in response, the expression of ACE2 increases, and the level of Ang II decreases relatively or absolutely. Since blood pressure is positively correlated with shear stress, the shear stress decreases as the blood pressure gets lower. Previous study shows that shear stress increases endothelial HA synthase 2 and HA synthesis to increase the thickness of glycocalyx and thus resist inflammation [94]. Also, in vitro studies have shown that the expression of hyaluronate synthase 2 mRNA and protein is temporarily upregulated by the phosphatidylinositol 3-kinase-Akt pathway in response to shear force changes in venous EC exposed to continuous or pulsatile shear forces [94]. Low shear stress inhibits eNOS-Ser-633 phosphorylation and, at least partially, NO production by activating hyaluronidase 2 to degrade HA in the glycocalyx [95]. Here comes the result that lower shear stress causes a decrease in thickness of glycocalyx, and inflammation is promoted. Diabetic patients exhibited a clinical phenomenon indicating preexisting damage to the endothelial glycocalyx. Additionally, the confirmation of heightened inflammation through the widespread migration of inflammatory cells further supports this notion [96]. Since spike protein interacts with ACE2, intracellular ROS levels have been upregulated. Excessive ROS hinders the PI3K/AKT/mTOR pathway, thereby enhancing the autophagic response. As a result, autophagy induced by SCV-2-S triggers inflammatory reactions and apoptosis in infected cells [97]. In addition, disruption of redox homeostasis forces nonimmune cells to synthesize ROS, which depolymerizes and destroys the structure of HS, CS, and HA GAG [98]. For example, HA is degraded in certain disease states by hyaluronidases and ROS to generate fragments with a reduced molecular weight. (<500 kDa) [99]. It indicates that the glycocalyx structure gets damaged, and glycocalyx constituents diffuse into the bloodstream [100]. According to clinical experiments, plasma from COVID-19 patients promotes glycocalyx shedding and redox imbalance in ECs [101]. In addition, ROS and proinflammatory cytokines, such as TNF-*α*, IL-1*β*, and IL-6, activated sheddases, including heparanase, matrix metalloproteases, and hyaluronidase to induce glycocalyx degradation [101].

**Figure 1:**
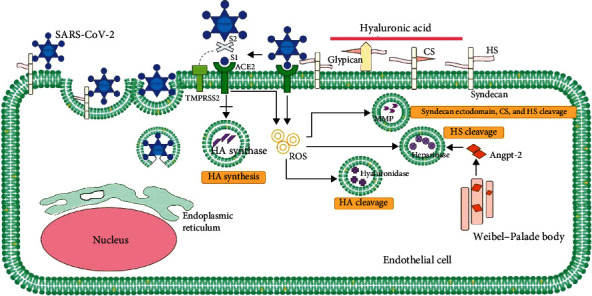
Schematic representation of the process of SARS-CoV-2 invasion through endothelial glycocalyx. The invasion of SARS-CoV-2 to endothelial cells disrupts glycocalyx. SARS-CoV-2 exploits HS to facilitate the attachment of spike-bearing viral particles to the cell surface through the glycocalyx. S1 of SARS-CoV-2 binds with ACE2, which suppresses the amount of HA synthase and promotes the production of ROS. ROS activates glycocalyx sheddases, including hyaluronidases, MMPs, and heparinases. Hyaluronidases cleave HA. MMPs cleave syndecan ectodomain, CS, and HS. Heparinases cleave HS. In addition, SARS-CoV-2 infection increases the level of Angpt-2, which activates heparanase release, consequently leading to degradation of the endothelial glycocalyx. HA, hyaluronic acid; HS, heparan sulfate (HS); CS, chondroitin sulfate. ROS, reactive oxygen species; MMP, matrix metalloproteases.

The spike protein of the coronavirus specifically binds to ACE2, and subsequently, as with SARS-CoV, SARS-CoV-2 has been shown to activate the ACE2-mediated endocytosis signaling pathway [102]. On the surface of EC, TMPRSS2 coexpress with ACE2, facilitating viral entry into target cells [103]. Spike ectodomain is cleaved into peripheral S1 and integral S2. The S protein, a class I fusion protein, is trimeric in nature and exists in a metastable prefusion conformation, which undergoes significant structural changes to facilitate the fusion of the viral membrane with the host cell membrane. The initiation of this process occurs when the S1 subunit attaches to a receptor on the host cell. The connection with the receptor disrupts the prefusion trimer, leading to the detachment of the S1 subunit and causing the S2 subunit to transition into a stable postfusion conformation [87]. The surface unit S1 from S protein binds to ACE2, contributing to the viral attachment to the surface of ECs. TMPRSS2 cleaves S protein at the S1/S2 and the S2′ site to prime S protein [49]. Furthermore, working synergistically with TMPRSS2, virus entry could also rely on the functioning of endosomal/lysosomal cysteine proteases cathepsin B, L (CTSB, CTSL), albeit their effectiveness being lower than that of TMPRSS2 [104]. The binding of ACE2 and S1 launches endocytosis, then clathrin-mediated vesicles form, SARS-CoV-2 enters into host cells [105]. The actin cytoskeleton polymerization is triggered by the endocytosis process, involving actin fibers in various stages of internalization, endosomal sorting, and trafficking of viral particles in ECs [106]. Host cell defense starts from binding of the SARS viral spike protein to ACE2 and downregulates ACE2 by triggering enzyme Internalization and proteolytic shedding of its extracellular domain [107]. The shed of S1 activates the S2 subdomain. HR-1 and HR-2 are heptad-repeat regions located in the S2 region. With the heptad-repeat regions collapsing into coiled coils, the cellular and viral membranes are brought into close proximity [108]. The conformational changes of the HR-1 and HR-2 induce the creation of an oligomeric structure, resulting in coalescence between the viral and target cell membranes [107]. It produces a pore, allowing the RNA and RNA-associated nucleocapsid proteins to have access to the cellular cytosol, escaping immune system surveillance and clearance, then infection starts (Figure 1).

Additionally, abnormal granule secretion was observed in infected ECs. Clinical data shows that both sTie2 [64] and angiopoietin-2 (Angpt-2) [109] levels are elevated in COVID-19 patients. The severity of the disease is correlated with an elevated level of Angpt-2 [110]. Weibel–Palade bodies are the storage granules of ECs. ECs getting infected activates Weibel–Palade body exocytosis. Weibel–Palade bodies compartments' exocytosis releases angiopoietin-2. Then, the glycocalyx gets disrupted through two kinds of paths. On the one hand, it has been shown that Angpt-2 activates heparanase release from the endothelium; consequently, it leads to enzymatic degradation of the endothelial glycocalyx [111]. On the other hand, glycocalyx self-protection is weakened. Tie2 works as a known glycocalyx-maintaining factor. The functional role of Ang-2 is well-established as an antagonist of Ang-1-Tie2 signaling. Mechanically, it plays the putative role in the transcriptional regulation of heparanase [112]. So, Ang-2 antagonizes Tie2 protection to weaken the glycocalyx structure.

Based on the map of protein–protein interactions between the SARS-CoV-2 virus and human proteins, 332 human proteins interact with 27 viral proteins [113]. Around 13% of host proteins show significant differences in phosphorylation upon infection. Interactions between the virus and host proteins bring about alterations in phosphorylation either by influencing the subcellular localization of host proteins or physically obstructing kinase access. In addition, the phosphorylation of these proteins during infection could indicate an extra method of functional regulation concerning the potential factors that may affect dependency and restriction [114].

Pre-viral RNA is protected by N protein, RNP complex, and nsp13. Within the virus particle, the N protein is situated and interacts with viral RNA [115]. Its primary function involves safeguarding genomic RNA by creating the RNP complex, which then undergoes condensation through the interaction with the M protein [116]. SARS-CoV-2 relies on its abilities to repurpose host RNA-binding proteins (RBPs) and to evade antiviral RBPs [117]. Nsp13 Helicase serves as a versatile enzyme engaged in both genome unwinding and the initial stage of mRNA capping. The cap structure protects nascent mRNAs at their 5′ ends, which makes viral mRNA more stable and able to evade the host immune response [118].

At the beginning of virus proliferation, SARS-CoV-2 forces the host cell to put viral RNA higher prioity compared to host cell RNA. Nsp13 interaction with Pol *δ* results in a cell cycle arrest in the S-phase to suppress host DNA replication. During replication, the SARS-CoV-2 N protein engages with various RNA-processing proteins, such as LARP1 and RRP9, which undergo distinct phosphorylation patterns during infection. LARP1 undergoes reduced phosphorylation at multiple sites, which in turn enhances its binding affinity for the 3′ untranslated regions of mRNAs that encode ribosomal proteins. As a result, this process actively leads to the inhibition of protein synthesis [119]. Following translation, the N protein undergoes phosphorylation, a crucial process that enables distinguishing between viral and nonviral mRNA binding. This implies a diverse impact on RNA regulation, indicating a pleiotropic effect [120]. The NUP98/RAE complex binds to ORF6, resulting in an elevation in NUP98 phosphorylation at S888, a site located within its peptidase domain. For proper localization to the nuclear pore, NUP98 undergoes autocatalytic cleavage. Therefore, it is likely that the binding of NUP98 with ORF6 and/or its virus-induced phosphorylation could impede host mRNA export through the nuclear pore [121, 122]. Then comes viral RNA replication. In response to SARS-CoV-2 infection, there is a halt in the cell cycle at the S/G2 phase, which serves the purpose of ensuring an ample supply of nucleotides. This arrest also facilitates the movement of crucial cellular components from the host nucleus to the replication site in the cytoplasm, favoring viral replication. During infection, a significant number of protein interactors of Nsp12 exhibited reduced phosphorylation. Nsp12 encodes the RNA-dependent RNA polymerase, which plays a crucial role in the replication of the viral genome. Notably, some of these interacting proteins, like LARP4B and CRTC3, are associated with RNA processing. The regulation of these interactions might have functional consequences for Nsp12 in replicating the viral genome. Nsp8 establishes interactions with multiple proteins, leading to an increase in phosphorylation levels for LARP7 and MPHOSPH10, while CCDC86 experiences a decrease in phosphorylation at various sites. Particularly noteworthy, LARP7 and MEPCE play crucial roles as regulators of RNA polymerase II-mediated transcription elongation within the 7SK small nuclear ribonucleoprotein particle (snRNP) complex. The control of these phosphorylation sites could have an impact on the regulation of the positive transcription elongation factor b (P-TEFb [CDK9]) and, consequently, the transcriptional regulation of the virus [114]. Viruses have the ability to exploit the ubiquitin system, utilizing it to augment multiple stages of the replication cycle and intensify pathogenesis [123]. SARS-CoV's PLpro protein acts as a DUB enzyme essential for the cleavage of viral polyproteins, leading to the formation of a functional replicase complex that facilitates viral dissemination [124]. According to certain reports, blocking the activity of PLpro hinders the replication of both SARS-CoV and SARS-CoV-2 [125]. DDX1 is involved in modulating viral RNA transcription by promoting the production of extended viral RNAs [126]. Furthermore, apart from the activation of DDR pathways due to the accumulation of host DNA damage, it can also have a favorable impact on the replication of viral RNA genomes. To prepare for virus assembling, mRNA is produced. Nsp14, an essential enzyme in mRNA proofreading and final capping, plays a pivotal role. Specifically, the ExoN domain of Nsp14 is responsible for hydrolyzing both single- and double-stranded RNAs, making it a crucial component for the self-correction of coronaviruses [127]. DDX5 plays a pro-viral role in coronavirus infection by interacting with Nsp13. This implies that the host helicase might serve as a coactivator, boosting viral genome transcription and promoting virus proliferation [128]. Using the host's ribosome, ORF1a and ORF1b translate 16 NSPs (nsp1–nsp16) that are required for viral RNA synthesis [129]. Positive ssRNA templating, RNA polymerase catalyzes to synthesize negative ssRNA. Also, positive ssRNA templates are used for the synthesis of structural proteins for progeny viruses, including the spike protein, envelope protein, membrane protein, and nucleocapsid protein [130]. After being assembled by material which is from the host cell, the progeny virus leaves the host cell. Furthermore, infection triggers a notable increase in filopodial protrusions containing CK2, and these protrusions exhibit budding viral particles. It appears that the viral particles are emerging from these protrusions. In the host cell, due to the discrimination against host cell DNA, the host DNA repair mechanism is attacked. On the one hand, infection with SARS-CoV-2 induces DNA damage and triggers a modified response to DNA damage.The degradation of CHK1, orchestrated by viral factors ORF6 and NSP13, through the proteasome and autophagy pathways, leads to DNA damage. When CHK1 is depleted, it results in the loss of RRM2, an essential element of the ribonucleotide reductase complex. As a consequence, there is a shortage of deoxynucleoside triphosphates, leading to hindered progression through the S-phase and accumulation of DNA damage [131]. Reduced import of host RBPs into the nucleus during SARS-CoV-2 infection may lead to the formation of R-loops. During the advanced phase of infection, there is a potential for the accumulation of R-loops within the cell, leading to the overwhelming of the DNA repair machinery, ultimately resulting in DNA damage. Moreover, the SARS-CoV-2 N protein interferes with the recruitment of 53BP1 at double-strand breaks (DSBs) by competing with dilncRNAs for binding, thereby obstructing the process of DNA repair [132]. On the other hand, according to computational modeling, it was discovered that the spike protein subunit 2 of SARS-CoV-2 has the potential to interact with BRCA1. Based on bioinformatic analysis, it was indicated that the S2 subunit of SARS-CoV-2 exhibits robust interactions with P53, breast cancer type 1 susceptibility protein (BRCA-1), and breast cancer type 2 susceptibility protein [133]. BRCA proteins participate in HR to repair DNA DSBs [134]. That indicates SARS-CoV-2 infection participates in host DNA repair, requiring further studies. In situations where the DNA damage response fails to address prolonged and irreparable DNA lesions, damaged DNA is secreted into the cytoplasm, which triggers activation of the cGAS-STING pathway [135]. Innate immune receptors will quickly identify any cellular changes and trigger or intensify inflammatory responses, aimed at eliminating potentially cancerous cells and thereby preventing the continuation of DNA damage [136]. Moreover, COVID-19-induced DNA damage may have an impact on the circadian rhythm and telomere shortening, hastening the process of epigenetic aging [137]. Concerning host cell, SARS-CoV-2 infection promotes casein kinase II (CK2) and p38 MAPK activation, then promotes the shutdown of mitotic kinases, resulting in cell cycle arrest [114]. Using host cholesterol, virus assembles cholesterol to its spike. Indeed, infection leads to the presentation of fusogenic viral proteins on the plasma membrane of the host cell. As a result, neighboring cells have the ability to fuze together, forming multinucleated syncytia. Cell–cell fusion plays a key role in viral replication or evasion of the host immune response [138]. Once the virus is released, the body's immune cells detect it through the pattern-recognition receptor. This prompts a cascade of signal transduction events, leading to the release of numerous cytokines. These cytokines, in turn, activate more immune cells, encouraging their involvement in the virus elimination process. As a result, an increasing number of immune cells and cytokines gather to combat the virus. A strong immune response is locally initiated. Furthermore, when the glycocalyx is broken down, soluble HS is released, acting as a danger-associated molecular pattern and triggering an inflammatory burst through interaction with Toll-like receptors, which forms an integral part of innate immunity [139].

However, in some cases, uncontrollable positive feedback takes the place of ordered negative feedback; thus, uncontrolled and overly aggressive immune response results in immune-related harm to the human body. It triggers a cytokine storm and mediates widespread lung inflammation [56]. The virus replicates to high titers very early after infection, which leads to an increased cytopathic effect and results in elevated levels of proinflammatory cytokines and chemokines released via infected ECs [140]. As a result, these cytokines and chemokines coordinate a significant influx of inflammatory cells into the lungs [141]. Noninfected cells also respond to viral infection [142]. The inflammation linked to a cytokine storm originates from a specific site and then disseminates throughout the body through systemic circulation, where its main component is cytokines. Cytokines work together, depend, and enhance each other, building complex nonlinear networks. After the immune system recognizes viral RNA, there launches a signaling cascade; infected cells and immune cell produce about 15 cytokines to defend virus, known for increased interleukin (IL)-6, IL-2, IL-7, granulocyte-colony stimulating factor, interferon-gamma (IFN-*γ*) inducible protein 10, monocyte chemoattractant protein 1, macrophage inflammatory protein 1-alpha, and tumor necrosis factor-alpha (TNF-*α*) [56]. Additionally, in cytokine storms, elevated levels of crucial cytokines such as IFN-*γ*, IL-1, IL-6, TNF, and IL-18 play pivotal immunopathological roles [143].

Inflammatory mediators TNF-alpha and IL-1 trigger an inflammatory cascade process through regulating proinflammatory signals. Intracellular kinase and extracellular signal-regulated kinase leads to different levels of proinflammatory cytokine expression. Research has shown that applying TNF-alpha causes substantial changes in the permeation characteristics of the endothelial glycocalyx, regardless of leukocyte adhesion. This finding reinforces the notion that the glycocalyx plays a crucial role in cytokine-induced damage [19]. Furthermore, the proinflammatory cytokine TNF-alpha mediates the shedding of heparin sulfate, the main component of PG, and SDC-1, the backbone of the glycoprotein network, from glycocalyx [144]. According to previous research, the secretion of NOX induced by the proinflammatory cytokine TNF-alpha also played a role in causing oxidative stress and endothelial dysfunction at the local level during COVID-19, thus providing an explanation for the observed outcomes [145].

Cytokine storm disorders encompass a complicated and interlinked network of cell types, signaling pathways, and cytokines. Cells are activated in a receptor-independent and cytokine-dependent manner. These cells are capable of triggering or propagating more cytokines. For example, TNF-alpha induces the upregulation of IFN-*γ*, leading to a synergistic effect that enhances the inflammatory response [146]. Moreover, endothelial activation by TNF-alpha upregulates the biosynthesis of IL-1 [147]. Colony-stimulating factors enhance the population of cytokine-producing macrophages in the region of local inflammation. CSF is additionally involved in an interconnected proinflammatory cytokine network that comprises IL-1 and TNF, working together in a mutually dependent manner [148]. Cytokines storm increases the permeability of vessel wall and then damages the structure. The impair leads to abundant tissue factor release from ECs, then activates the extrinsic pathway of blood coagulation [149]. When the negative charged under sulfated glycocalyx contact with the bloodstream, factor XII turns into XIIa, so the intrinsic pathway of blood coagulation is activated. Massive TNF and IL-1 suppress the generation of TM and activated protein C to reduce the suppression of blood coagulation [150]. Also, TNF and IL-1 reduce tissue plasminogen activator but increase tissue plasminogen activator inhibitor. Finally, fibrinolysin's generation and function are decreased [151].

Evidence indicates that the endothelial glycocalyx layer (EGL) undergoes notable alterations in its characteristics during inflammatory conditions, such as partial degradation [152]. This potentially makes the endothelium more susceptible to blood components' exposure and facilitate continuous rolling and adhesion of leukocytes. Since IL-6 activates immune cells (T cells and macrophages), immune cells are recruited to the incomplete EGL to release antibodies and complement, clearing cells infected with the virus. Recent studies have shown that antibodies and complement activate ECs, leading to cell division and release of heparin sulfate. Antibody and complement fragment C5a mediate the release and loss of heparin sulfate in ECs [153]. In addition, Type I and Type III IFN affect viral RNA synthesis. ACE2 mRNA is upregulated in response to IFN stimulation [154]. T cells are a major source of cytokines and chemokines. When T cells are activated and differentiated, they secrete a second wave of cytokines, of which IFN-*γ* is the main force. At this point, cytokine levels exceed the desired level, leading to an excessive accumulation of inflammatory cells, including monocyte-macrophages, T cells, and so on. As pathogenic inflammatory monocyte-macrophages accumulate, it leads to an increased production of cytokines in the lungs [141]. When a large number of inflammatory cells accumulate in the alveoli, a large number of normal cells are damaged, and oxygenation decreases, resulting in lethal respiratory failure.

In terms of severity, lung is the primary organ targeted by SARS-CoV-2 [56, 155]. The majority of individuals with COVID-19 exhibit lung manifestations only, while several severe patients present with other systems' symptoms or even multiple organ dysfunction syndrome. First, except lung, ACE2 is predominantly found in nasal epithelial cells, type II alveolar epithelial cells, esophagus, colon, ileum, cornea, gallbladder, and common duct, among other locations, as reported [156]. This indicates that all organs expressing ACE2 are at risk of infection. Second, the release of cytokines during a storm enters the bloodstream and leads to damage in distant organs by activating their intrinsic inflammatory cells. Moreover, the presence of circulating cytokines causes widespread injury to the endothelium in various organs. Subsequently, circulating inflammatory mediators establish a positive feedback loop, interconnecting organ dysfunctions [157]. Also, according to COVID-19 patients' multiorgan proteomic profiling, multiple organs displayed evidence of systemic hyperinflammation and dysregulation in glucose and fatty acid metabolism. Dysregulation of key factors involved in angiogenesis, hypoxia, fibrosis, and blood coagulation were also observed in multiple organs [158].

## 5. Clinical Therapy

Given EGL's involvement in adhesion, permeability, and coagulation, an intriguing therapeutic approach for COVID-19 and its complications could involve repairing already damaged EGLs and safeguarding them from further harm.

### 5.1. Virus-ACE2 Noncompetitive Antagonist Treatment

The binding between RBD and ACE2 is reduced by naturally occurring and clinically accessible triterpenoids like oleanolic acids and glycyrrhetinic. Additionally, primary and secondary bile acids, along with their amidated derivatives, as well as semisynthetic derivatives, also contribute to the reduction of RBD/ACE2 binding [159]. In many studies, ursodeoxycholic acid inhibited the proinflammatory cytokines, such asTNF-*α*, IL-1*β*, IL-2, IL-4, and IL-6 at both mRNA and protein levels to prohibit cytokine storm [160, 161].

### 5.2. Virus-ACE2 Competitive Antagonist Treatment

It has been reported that the levels of ACE2-expressing circulating extracellular vesicles (EVs) in the plasma of COVID-19 patients rise, and these elevated levels are linked to severe disease progression. When EVs expressing ACE2, obtained from human plasma or cells, are isolated, it exhibits a neutralizing effect on SARS-CoV-2 infection by competing with cellular ACE2 [162].

### 5.3. Anticoagulant Treatment

According to reports, there is evidence pointing to a favorable impact of heparin/low molecular weight heparin usage on COVID-19 mortality rates. It neutralizes chemokines and cytokines. It reduces cytokines storm to protect glycocalyx. It inhibits heparanase activity, maintaining the thickness of glycocalyx [163]. Additionally, in vitro investigations with ECs revealed a significant stimulation of HS biosynthesis on the cell surface in response to Hep, which takes place immediately after the ECs are exposed to Hep [164].

### 5.4. Analog Treatment

Sulodexide is a sulfated polysaccharide complex derived from mammalian intestinal mucosa, comprising 4/5 of HS and 1/5 of DS [165]. Sulodexide maintains the function of the endothelial glycocalyx by promoting the synthesis of GAG and reducing its breakdown [166].

### 5.5. Hormone

Including dexamethasone, glucocorticoids have been reported to suppress the expression of inflammatory mediators to forbid activating neutrophils [167]. Hydrocortisone stabilizes the endothelial glycocalyx by suppressing TNF-*α*, allowing the preservation of the physiological endothelial permeability barrier despite facing inflammatory challenges [168].

### 5.6. Cytokines Treatment

Cytokine inhibitors, including IL-6 and IL-1 blockade and immunosuppressants, decrease the amount of cytokine to suppress cytokine storm [169]. The IL-1 receptor antagonist Anakinra is under promising assessment in randomized clinical trails [170]. JAK-STAT inhibitors block the pathway and also inhibit IL-6 and IFNs [171]. JAK inhibitor tofacitinib plays roles in vitro and in vivo [172].

### 5.7. Complement System Therapy

Now, Eculizumab, a humanized monoclonal antibody targeting C5, stands as the most frequently employed complement inhibitor in clinical settings. Besides that, ravulizumab is a recombinant humanized anti-C5 monoclonal antibody. IFX-1 blocks the effect of C5a without interfering with C5b's function and keeping the membrane attack complex whole. Avdoralimab is an IgG1-kappa anti-C5aR1 blocking antibody. The MASP-2 inhibitor narsoplimab (OMS721) is applied to disturb the interaction between MASP-2 and the *N*-protein of SARS-CoV-2 [173]. C-reactive protein (CRP) disposes of the bacteria and host cells undergoing apoptosis or necrosis as the complement components of C1q-C4's work [174]. As high CRP levels are along with COVID-19, lowering CRP levels by therapeutic apheresis potentially reduces the pathological progression in the early stage [175].

### 5.8. Stem Cell Therapy

Mesenchymal stem cells (MSCs) suppress the release of proinflammatory cytokines such as IL-6, IL-12, IL-1*α*, TNF-*α*, and IFN-*γ*, thereby decreasing the frequency of cytokine storms [176]. MSC also secretes vascular endothelial growth factor and keratinocyte growth factor to relieve ARDS and regenerate injured lung tissues [177].

### 5.9. Physical Method

Researchers explored the importance of the cytokine removal by means of two resin membranes (HA330 and Mediasorb) in COVID-19 patients treated in ICUs. Although considering from pathophysiological basis, the possibility of utilizing cytokine adsorption techniques to modulate the immune response in critically ill COVID-19 patients is achievable. It is too early to assert good about the result [178]. The CytoSorb is a hemoadsorption column designed to eliminate inflammatory mediators from the circulation [179]. Artificial liver technology wipes out inflammatory factors on a large scale. Based on the previous use of artificial liver technology on H7N9 bird flu, it is being used in COVID-19 with progress now [180, 181].

## 6. Conclusion

The glycocalyx that covers the vascular endothelium is a hair-like network of proteins and polysaccharides. It performs a rich function and is physiologically and pathologically important, playing a role in protecting ECs from SARS-CoV-2. In the case of incomplete glycolyx, it contributes to serious infections. Based on previous research, glycocalyx has been shown to be a potential tool for the treatment and prevention of COVID-19. Although various treatments have been shown to be effective in theory, clinical practice is still ongoing. At the same time, more therapeutic targets need to be explored, and multitarget therapy has great prospects.

## Data Availability

Data availability is not applicable to this article as no new data were created or analyzed in this study.
